# Comparative Evaluation of Adaptation of Esthetic Prefabricated Fiberglass and CAD/CAM Crowns for Primary Teeth: Microcomputed Tomography Analysis

**DOI:** 10.1155/2021/1011661

**Published:** 2021-09-26

**Authors:** Ece Irem Oguz, Tuğba Bezgin, Ayse Isıl Orhan, Kaan Orhan

**Affiliations:** ^1^Department of Prosthodontics, Faculty of Dentistry, Ankara University, Ankara 06500, Turkey; ^2^Department of Paediatric Dentistry, Faculty of Dentistry, Ankara University, Ankara 06500, Turkey; ^3^Department of Paediatric Dentistry, Faculty of Dentistry, Ankara Yildirim Beyazit University, Ankara 06830, Turkey; ^4^Department of Dentomaxillofacial Radiology, Faculty of Dentistry, Ankara University, Ankara 06500, Turkey

## Abstract

Adaptation is an important factor for the clinical success of restorations. However, no studies are available evaluating the adaptation of primary crowns. The aim of this study was to compare the adaptation of crowns fabricated by CAD/CAM technology versus prefabricated fiberglass primary crowns. Typodont maxillary central, canine, and mandibular molar teeth were prepared to serve as master dies after the size of Figaro crowns was determined (*n* = 10). Master dies were scanned with an intraoral scanner, and 10 identical CAD/CAM crowns were fabricated from resin-ceramic blocks. Figaro and CAD/CAM crowns were placed on the corresponding master dies and scanned via micro-CT. Three-dimensional volumetric gap measurements were performed to evaluate the overall adaptation. A total of 255 location-based linear measurements were allocated into 4 categories: marginal, cervical-axial, middle-axial, and occlusal. Statistical analyses were performed with factorial ANOVA, repeated measure ANOVA, and LSD tests (*α* = 0.05). CAD/CAM crowns showed significantly lower overall and location-based gap measurements than Figaro crowns regardless of tooth number (*p* < 0.05). For all groups, mean marginal discrepancies were lower than occlusal measurements (*p* < 0.05). Both crown types showed higher marginal gaps for molar teeth than for canine and central incisors with no significant difference between them (*p* > 0.05). CAD/CAM-fabricated crowns showed better marginal and internal adaptation than prefabricated Figaro crowns.

## 1. Introduction

Early childhood caries (ECC) is defined as the presence of decay and decay-related filled or lost tooth surfaces in one or more teeth of children aged 71 months or younger [1, 2]. ECC begins with white lesions along the margin of the maxillary primary incisors and can progress rapidly, leading to the destruction of the crown [1, 3]. Besides esthetic, nutrition, and phonation problems, ECC may cause detrimental effects on general health [4]. If treatment for ECC is delayed, serious disorders such as pain dysfunction, negative effects on growth and development, psychological problems, and a decrease in quality of life may occur [1, 2, 5].

Depending on the progression of the disease, different treatment modalities for ECC can be applied from preventive techniques to crown restorations [4]. Primary teeth with widespread crown damage have been successfully treated with stainless steel crowns (SSCs) for many years [6, 7]. However, SSCs could not meet the esthetic expectations of paediatric patients and parents [8, 9]. Restorations that can satisfy increasing expectations have been obtained as a result of the developments in technology and esthetic material science for crowns in paediatric dentistry [8, 10, 11]. Veneered SSCs, composite strip crowns, and prefabricated zirconia crowns were the first materials introduced to accomplish an esthetic outcome [10–12]. The most preferred esthetic crown nowadays is prefabricated zirconia crowns which are available from different manufacturers. The most important advantage of zirconia crowns is that gingival and plaque indices are lower among these crowns than other crown types [8]. However, these crowns have certain disadvantages such as (i) they are very technique sensitive and (ii) they require excessive tooth preparation to provide a passive fit [12, 13].

One of the newly launched materials to overcome such disadvantages is Figaro crowns made of fiberglass [14]. They are tooth colored and require less tooth reduction than paediatric preformed zirconia crowns with its flex-fit technology [14]. It is less technique sensitive than both composite strip crowns and zirconia crowns with a similar technique to place a SSC [15]. However, a previous study indicated failures in terms of crown retention, fracture resistance, and color deterioration for Figaro crowns compared to SSCs after 6 months of clinical evaluation period [14].

Another method of note to achieve esthetics in paediatric dentistry is the computer-aided design and computer-aided manufacturing (CAD/CAM) technology. Developments in CAD/CAM technics have enabled the production of esthetic and functional restorations for both permanent and primary dentition [16]. Customized crowns can be manufactured chairside by using CAD/CAM in a single appointment. Among a wide variety of blocks available for CAD/CAM, resin-ceramic blocks stand out with advantageous features for primary dentition including the wear prevention of opposing dentition due to their low hardness values and absorption of functional stresses because of their low modulus of elasticity [17, 18]. Another beneficial outcome of low modulus of elasticity was reported as the accurate adaptation of the restoration [18].

The marginal and internal adaptations are critical factors that determine the success of the restoration. While the marginal misfit was related to cement dissolution, microleakage, plaque accumulation, secondary caries, and periodontal disease, the internal misfit was associated with poor mechanical retention and reduced fractural strength [7, 18–20]. The adaptation may vary depending on the restorative material or production method of the restoration [20, 21]. No study to date has focused on the adaptation of crowns applied on primary teeth.

Therefore, the present in vitro study was aimed at comparing the adaptation of two types of esthetic paediatric crowns, the prefabricated fiberglass and custom-made resin-ceramic crowns, for primary teeth. The null hypothesis tested was that prefabricated fiberglass and CAD/CAM crowns would not differ in terms of adaptation.

## 2. Materials and Methods

This study has followed the CRIS guidelines for in vitro studies as discussed in the 2014 concept note.

### 2.1. Master Die Preparation

The marginal and internal adaptations of CAD/CAM crowns and fiberglass primary crowns were compared by using microcomputed tomography. A sample size of 10 per group was determined based on a power analysis (expected difference = 0.01, standard error of the mean = 23.85, *α* = 0.05, 1 − *β* = 0.8) [21]; 10 identical fiberglass crowns (Figaro crowns, Size XS; Figaro Crowns Inc., Minnesota, US) were selected considering the size of typodont primary central incisor (#51), canine (#53), and molar (#75) teeth prior to preparations as suggested by the manufacturer (*n* = 10). Typodont teeth were placed on a typodont model (Frasaco Dental Model, AK-6; Frasaco GmbH, Tettnang, Germany) and prepared by the same operator (TB) according to the manufacturer's preparation guide and suggestions for Figaro crowns [22]. The finished preparations and seating of the chosen Figaro crowns were approved by 2 operators (EİO and TB). The margin lines of the 3 master dies were marked by using a permanent marker.

### 2.2. CAD/CAM Process

The prepared #51, #53, and #75 master dies were placed on the typodont model one by one and digitized with an intraoral scanner (CEREC Omnicam; Dentsply Sirona, York, US). To replicate the external form of fiberglass crowns, the “biocopy” tool of the CEREC software (SW 4.6, Dentsply Sirona) was used. For this purpose, prefabricated fiberglass crowns were placed on the corresponding master dies and scanned with the CEREC Omnicam. The scanning process took approximately 5 min for each tooth. Preparation margins were drawn by the “automatic margin finder” tool, and deviations from the marked margin line were corrected manually. The die spacer parameter was set as 120 *μ*m for all teeth, and the software automatically designed virtual crowns based on the scans of the fiberglass crowns. Ten CAD/CAM crowns for each master die (#51, #53, and #75) were milled from resin-ceramic blocks (CERASMART 270; GC Dental Products, Tokyo, Japan) by using a clinical type milling unit (CEREC MC XL; Dentsply Sirona) (*N* = 30). The milling time of each crown was about 10 min.

The sample size and test groups of the study are presented in Table 1.

### 2.3. Micro-CT Evaluation

Figaro and CAD/CAM crowns were placed on the corresponding master dies one by one with finger pressure until complete seating, maintained in that position under an axial load of 5 kg for 10 min in a seating pressure device, and were fixed with a parafilm (Parafilm M film; Bemis Company, Inc., Oshkosh, WI, US). The master dies were scanned with and without crowns by using a high-resolution desktop micro-CT (Bruker Skyscan 1275, Kontich, Belgium). Each stabilized specimen was positioned perpendicularly to the X-ray beam to ensure standardized positioning in the scanning tube and scanned with the following conditions: beam current at 100 kVp, 100 mA, 0.5 mm Al/Cu filter, 10.1 *μ*m pixel size, rotation at 0.5 step, and 360° within an integration time of 10 min. The mean scanning time for each specimen was about 1 hour. Air calibration of the detector was done before each scan to minimize the ring artifacts. Beam-hardening correction and input of optimal contrast limits according to the manufacturer's instructions were carried out based on the former scanning and reconstruction.

Visualization and quantitative measurements were utilized by using NRecon (ver. 1.6.10.4, SkyScan, Kontich, Belgium), DataViewer (version 1.5.6.2, SkyScan), and CTAn (version 1.17.7.2, SkyScan) software. For the reconstruction parameters, ring artifact correction and smoothing were fixed at zero, and the beam artifact correction was set at 30%. First, the reconstructed images were superimposed with the DataViewer software. The scans of the master die alone were used as a reference for the standardization of the measurement points. The master die images without a crown (reference) and with a crown (target) were superimposed, generating a volume of subtracting image. This image represented the entire area and volume of the gap between the crown and the master die. Then, the CTAn software was used for the 3-dimensional (3D) volumetric gap measurements (mm^3^) to evaluate the overall adaptation.

**Figure 1:**
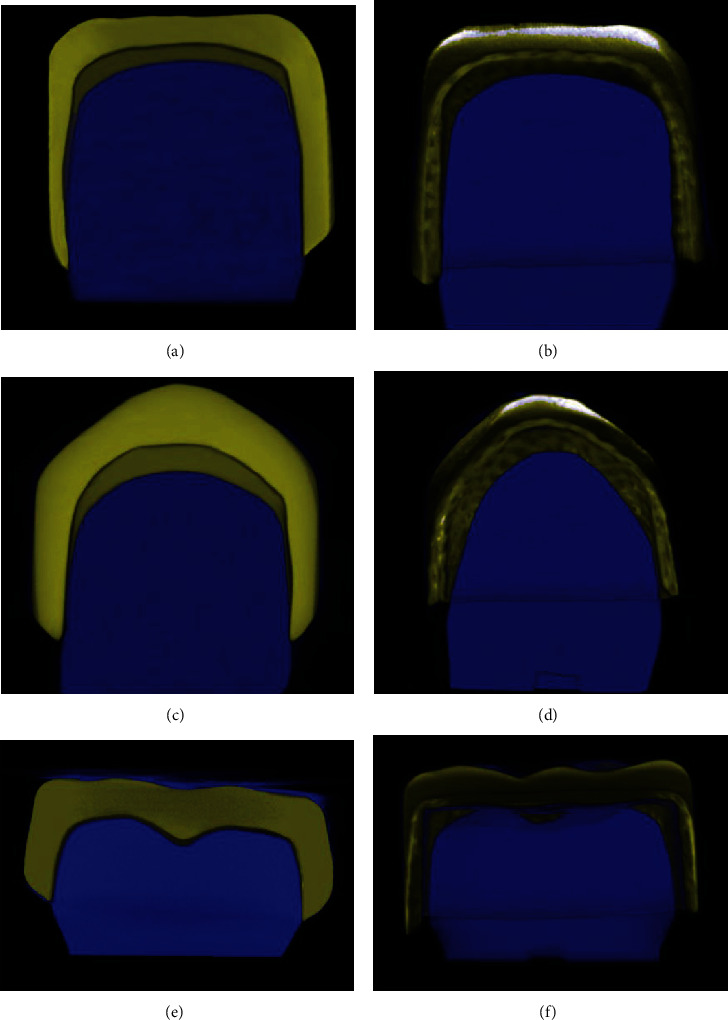
Representative micro-CT images of crowns applied on the corresponding dies. (a) CAD/CAM crown for #51; (b) Figaro crown for #51; (c) CAD/CAM crown for #53; (d) Figaro crown for #53; (e) CAD/CAM crown for #75; (f) Figaro crown for #75.

A semiautomatic global thresholding (binarization) process was applied with CTAn software to distinguish the gap from other structures by processing the range of grey levels and to obtain imposed images of black and white pixels only. In this procedure, a Gaussian low-pass filter for noise reduction and an automatic segmentation threshold was used. Then, 5 fixed regions of interest (ROI) with the same dimensions (1.5 × 1.5 mm for the central and canine teeth and 2.0 × 2.0 mm for molar tooth) were determined separately for each master die and for each slice to include the crown entirely. Forty equidistant vertical cuts from axial images were made in the mesiodistal direction. This procedure ensured the standardization of the location-based measurements. Seventeen measurement points were determined, and 85 measurements were done from 5 predesignated ROIs. Moreover, the observer repeated the measurements for each point 3 times. The mean values of all measurements were noted and were included in the statistical analysis. The observer also performed the study twice with an interval of 2 weeks to detect intraobserver variability. In total, 255 measurements were done for each crown. These 2D linear measurements (*μ*m) were allocated into 4 location categories as follows: marginal (absolute marginal discrepancy: the average of the linear distances from the finish line of the preparation to the outer margin of the restoration) [21, 23], cervical-axial (the average of horizontal gap measurements performed in the cervical third of the axial walls), middle-axial (the average of horizontal gap measurements performed in the middle third of the axial walls), and incisal/occlusal discrepancies (the average of vertical gap measurements performed in the incisal/occlusal surface). The reconstructed images were also processed in SkyScan CTVox (ver. 3.3.0, SkyScan) for visualization (Figures 1(a)–1(f)).

### 2.4. Statistical Analysis

To assess intraobserver reliability, the Wilcoxon matched pairs signed rank test was used for repeated measurements. The mean values of these measurements were considered to be the final data. The normality of the data was verified using Shapiro-Wilk test (*p* > 0.05). The overall volumetric gap measurements were statistically analyzed with factorial analysis of variance (ANOVA) and least significant difference (LSD) tests. Location-based linear measurement data were evaluated with repeated measure ANOVA and LSD tests. The statistical analyses were performed using R v.3.5.3 (Microsoft Corporation, Redmond, WA, US) (*α* = 0.05).

## 3. Results

Repeated measurements indicated no significant intraobserver difference for the observer (*p* > 0.05). Overall intraobserver consistency was rated at 92.6% between the two measurements, and all measurements were found to be highly reproducible.

Factorial ANOVA results and descriptive statistics for overall gap measurements are shown in Tables 2 and 3, respectively. CAD/CAM crowns showed lower overall mean gaps than fiberglass crowns irrespective of the tooth number (*p* < 0.05). Both crown types showed the highest volumetric gap for #75 (*p* < 0.05). The lowest overall volumetric gap for fiberglass crowns was obtained for the central incisor (*p* < 0.05), whereas for CAD/CAM crowns, no statistical difference was found between central and canine incisors (*p* > 0.05).

Repeated ANOVA results for linear measurements showed that the interactions between tooth number, crown type, and measurement location were significant (*p* < 0.001) (Table 4). Considering the differences between the location-based measurements for a certain tooth and crown type (Table 5), all groups showed lower mean gap values for the margin than that for the occlusal surface (*p* < 0.05). Both crown types applied on #51 and CAD/CAM crowns applied on #53 showed similar mean values for the marginal discrepancy and cervical-axial location (*p* > 0.05), whereas the other groups showed lower gap measurements for marginal discrepancy than that for the cervical-axial location (*p* < 0.05). Regardless of the crown type, middle-axial and incisal gap measurements were comparable for #51 and #53 (*p* > 0.05). The highest gap measurement for both crown types was obtained for the occlusal surface of #75 (*p* < 0.05).

**Figure 2:**
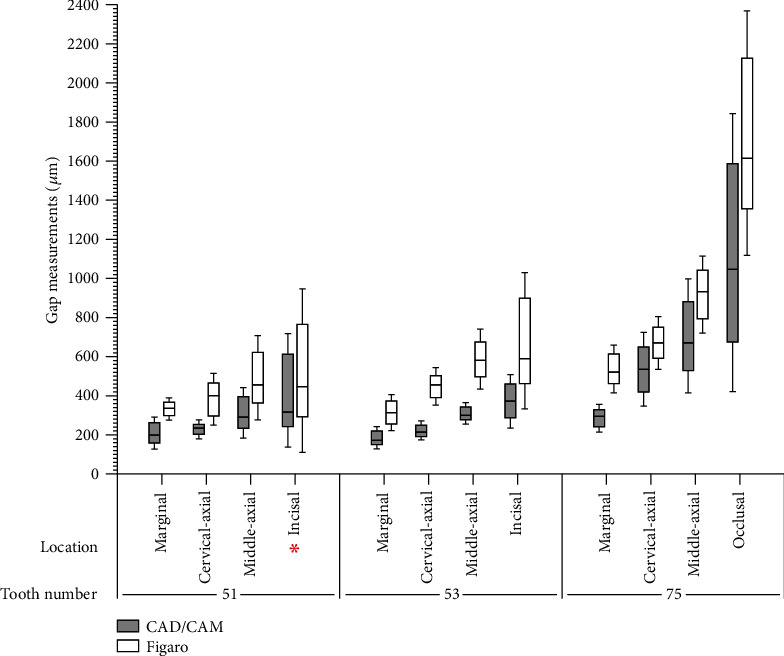
Comparison of gap measurements of different crown types based on location for each tooth. The asterisks (∗) indicate no statistical difference between groups (*p* > 0.05).

Gap measurements for CAD/CAM crowns were lower than fiberglass crowns regardless of the location and tooth number (*p* < 0.05) with an exception of #51 for which comparable incisal gap measurements were found for CAD/CAM and fiberglass crowns (*p* > 0.05) (Figure 2).

When the gap measurements obtained for different teeth were compared, #75 showed higher gap measurements than #51 and #53 irrespective of the location and for both crown types (*p* < 0.05). Considering fiberglass crowns, #53 showed higher middle-axial and occlusal gaps than #51 (*p* < 0.05). However, no significant differences were found between #51 and #53 at other locations either for CAD/CAM or for fiberglass crowns (*p* > 0.05).

## 4. Discussion

The adaptation of a crown is of importance for primary teeth as well as permanent dentition, considering that poor-fitting crowns may cause secondary caries or gingivitis [23]. This in vitro study compared the adaptation of CAD/CAM resin-ceramic and prefabricated fiberglass primary crowns by calculating overall, marginal, and internal gaps via micro-CT. The results showed significant differences between the gap measurements for CAD/CAM and fiberglass crowns concerning both overall and location-based evaluations. Therefore, the null hypothesis suggesting that the adaptation of fiberglass and resin-ceramic CAD/CAM crowns would be similar was rejected.

The adaptation can be evaluated by measuring the gap between the restoration and preparation with various methods such as direct microscopic measurement [18, 24], silicone replica technique [18], virtual seating of the crown and die by using their 3D scan data via reverse engineering software [20], and, as the newest technique, micro-CT imaging [21]. Precise linear 2D and volumetric 3D measurements can be performed in micron-level precision by using micro-CT, which was recommended as an innovative and nondestructive method for the in vitro evaluation of the adaptation [25]. Micro-CT allows for a great number of measurement points with close sectioning of the specimen, which ensures the reliability of the results [25]. In the present study, 5 ROIs were determined with equal sectioning in slices, and 255 measurements for each specimen were performed from 17 standardized points to ensure a comprehensive evaluation of internal and marginal adaptation of the crowns.

The present study compared the adaptation of two different esthetic crown types for primary teeth. Figaro crowns are composed of fiberglass, aramid, carbon, and quartz filaments embedded within a composite resin material [14]. The combination of these materials brings flexibility which enables a slight elastic deformation while placing the crown on the prepared tooth [26]. This flex-fit technology allows minimal tooth reduction, unlike zirconia esthetic crowns which require excessive preparation to compensate for the lack of flexibility [13, 14, 26]. To ensure the passive fit of zirconia crowns as recommended, retention and adaptation problems are frequently encountered [13]. Based on these considerations, zirconia esthetic crowns were not included in this study. SSCs are the most appropriate restorative materials in paediatric dentistry [8]. In addition, the tooth preparation is minimal, and their adaptation is flex-fit. However, SSCs were not included in this study as a control group because micro-CT does not allow the scanning of materials with high atomic number such as metals [27]. On the other side, CAD/CAM crowns were fabricated from resin-ceramic blocks considering the advantages for primary teeth and the similarity in composition to Figaro crowns. Therefore, custom-made CAD/CAM crowns were included as the control group.

In the present study, the overall adaptation was evaluated based on the 3D volumetric analysis and should be regarded as the total cement space [21, 28]. However, location-based linear 2D measurements provide data indicative of increased cement thickness at particular internal measurement points, as well as marginal adaptation [28]. CAD/CAM crowns showed better adaptation than Figaro crowns for both overall and location-based gap measurements and for all teeth. CAD/CAM crowns were designed based on the scans of Figaro crowns, and both crown types had identical outer forms. However, the internal contours were different as Figaro crowns have prefabricated, nonanatomical, and standardized inner surfaces while CAD/CAM crowns were custom-made. Micro-CT images for the mandibular molar showed that the CAD/CAM crown had rounded inner corners which were in harmony with the preparation outline (Figure 1(e)). On the other hand, right-angled internal corners that did not fit the preparation outline at the axioocclusal transition areas of the Figaro crown were observed (Figure 1(f)). Therefore, according to the present findings, it can be suggested that despite Figaro crowns allowing the restoration to adapt on the prepared tooth with flex-fitting, custom-made crowns fabricated with CAD/CAM technology provide better adaptation for primary teeth.

The uniformity of the gap between the preparation and the crown is important to ensure the retention form as well as fracture strength [21, 29]. Overall adaptation gives a general overview of the entire gap between the preparation and the crown; however, to evaluate the uniformity, location-based analysis is essential [21]. Previous studies reported that increased gap spaces at axial walls and the occlusal surface may reduce resistance to fracture [21, 29, 30]. Considering location-based adaptation, all groups showed a tendency for increased gap measurements from the marginal region to the occlusal surface. This finding corroborates with previous studies that reported the highest location-based gap measurements for the occlusal surface [31, 32]. For CAD/CAM restorations, the diameter and shape of the milling tools might limit the machining ability which would adversely affect the internal adaptation, especially at the occlusal surface [29]. On the other hand, frictional contacts that exceeded the flexibility limit of the Figaro crowns in the cervical region may have prevented proper fitting, resulting in an increased occlusal gap. Since disadvantages related to excessive occlusal gap include stress concentration and restoration fractures, clinicians should be cautious about occlusal adaptation when restoring primary teeth with Figaro or CAD/CAM crowns [19, 32].

Previous studies reported that the marginal gap values of ceramic crowns may range from 50 to 200 *μ*m [18, 20, 21]. Only CAD/CAM crowns fabricated for central and canine incisors were within these limits. The marginal gap for CAD/CAM molar crowns was above 200 *μ*m, yet lower than the marginal gap for Figaro crowns. All Figaro crowns exhibited marginal gap values exceeding the clinically acceptable range irrespective of tooth number. In the present study, the preparation design recommended for Figaro crowns was performed for all teeth, and optical impressions of the same prepared teeth were obtained to fabricate CAD/CAM crowns. Therefore, for both crown types, marginal adaptations were evaluated for knife-edge margin which was reported to result in the greatest marginal discrepancy compared to other margin designs [24]. Furthermore, the present study evaluated marginal adaptation based on the absolute marginal discrepancy which considers both the horizontal and vertical directions [20]. Marginal design and marginal adaptation evaluation method employed in the present study may be the reason for high marginal gap values. Furthermore, micro-CT images of the Figaro crowns showed an overextension in the outer margin line which would have increased the gap measurements for the absolute marginal discrepancy (Figures 1(b), 1(d), and 1(f)). Based on these findings, CAD/CAM crowns may be preferred over Figaro crowns considering the clinical significance of marginal adaptation.

In the present in vitro study, consistency of the results was ensured with standardized test conditions. Location-based gap measurements were performed by using the same ROIs and measurement points for all scans. To implement micro-CT measurements under the same conditions, CAD/CAM crowns were fabricated by scanning the same preparations on which Figaro crowns were adapted. Therefore, the preparations were standardized for both groups. Also, to eliminate differences in crown geometry, CAD/CAM crowns were designed based on the scans of Figaro crowns by utilizing the “biocopy” tool of the CEREC software. Nevertheless, limitations exist as in any in vitro study. Gap measurements were executed without cementation which may influence the fit of the restoration [24]. However, if the crowns were cemented on the corresponding master dies, the adaptation evaluation should have been performed on different preparations. To use a single standardized master die for each tooth, adaptation evaluation was performed without cementation. In addition, intraoral conditions such as soft tissue, saliva, and gingival fluid may affect the quality of the digital impression, thus adaptation. Further in vivo studies are warranted to evaluate the applicability of the CAD/CAM and Figaro crowns in paediatric dentistry and the effect of intraoral variables on the adaptation.

## 5. Conclusion

In this study, microcomputed tomography was first used to evaluate the adaptation of crowns for primary teeth, and the results showed that resin ceramic CAD/CAM crowns showed better overall, marginal, and internal adaptation compared to prefabricated fiberglass primary crowns for all primary teeth.

All crowns showed lower gap measurements at the marginal region compared to the occlusal surface, which is important for the clinical prognosis. A modality to define the clinically acceptable adaptation parameters for crowns applied to primary teeth can be developed based on the findings of this study.

## Figures and Tables

**Table 1:** Test groups of the study.

	Crown type	
Tooth number	CAD/CAM (*n*)	Prefabricated fiberglass (*n*)
51	10	10
53	10	10
75	10	10

**Table 2:** Factorial ANOVA results for overall gap measurements.

	SS	df	MS	*F*	*p* value
Tooth number	185.777	2	92.888	279.999	<.0001
Crown type	365.585	1	365.585	1102.003	<.0001
Tooth number∗crown type	39.124	2	19.562	58.967	<.0001

SS: sum of squares; df: degree of freedom; MS: mean squares.

**Table 3:** Mean and standard deviations (±SD) for overall gap measurements (mm^3^).

	Crown type	
Tooth number	CAD/CAM	Prefabricated fiberglass
51	3.08 (0.38)^Aa^	8.15 (0.74)^Ab^
53	2.93 (0.48)^Aa^	5.82 (0.61)^Bb^
75	5.15 (0.56)^Ba^	11.99 (0.61)^Cb^

Different superscript uppercase letters (A, B, C) in the same column and different superscript lowercase letters (a, b) in the same line indicate statistically significant difference (*p* < 0.05).

**Table 4:** Repeated measure ANOVA results for linear gap measurements.

	SS	df	MS	*F*	*p*
Tooth number	9859436	2	4929718	315.871	<.0001
Crown type	2867286	1	2867286	183.721	<.0001
Tooth number∗crown type	258328	2	129164	8.276	<.0001
Measurement location	6145911	3	2048637	255.888	<.0001
Measurement location∗tooth number	4239697	6	706616	88.261	<.0001
Measurement location∗crown type	150605	3	50202	6.271	<.0001
Measurement location∗tooth number∗crown type	474306	6	79051	9.874	<.0001

SS: sum of squares; df: degree of freedom; MS: mean squares.

**Table 5:** Mean and standard deviations (±SD) for location-based gap measurements (*μ*m).

Tooth number	Crown type	Location	Mean (SD)	Range
51	CAD/CAM	Marginal	197.8 (30.7)^A^	157.89-261.41
		Cervical-axial	235.9 (19.79)^AB^	199.80-257.1
		Middle-axial	297.03 (49.12)^BC^	230.15-393.29
		Incisal	356.11 (103.31)^C^	241.72-613.87
	Figaro	Marginal	336.53 (22.59)^A^	297.76-366.87
		Cervical-axial	400.44 (48.5)^AB^	295.27-464.57
		Middle-axial	454.01 (85.37)^B^	362.44-621.71
		Incisal	445.21 (180.73)^B^	288.36-763.2

53	CAD/CAM	Marginal	170.98 (22.11)^A^	148-218.21
		Cervical-axial	213.09 (17.87)^A^	189.52-249.88
		Middle-axial	301.44 (23.45)^B^	276.43-342.69
		Incisal	371.41 (50.91)^B^	284.39-459.88
	Figaro	Marginal	313.17 (32.39)^A^	252.95-373.85
		Cervical-axial	454.26 (40.31)^B^	386.98-501.17
		Middle-axial	580.43 (64.71)^C^	496.07-676.08
		Incisal	590.84 (129.51)^C^	461.73-897.04

75	CAD/CAM	Marginal	295.17 (26.48)^A^	241.77-327.34
		Cervical-axial	538.55 (74.31)^B^	418.21-651.8
		Middle-axial	672.18 (115.99)^C^	533.05-881.19
		Occlusal	1043.47 (254.81)^D^	673.32-1589.18
	Figaro	Marginal	520.55 (47.55)^A^	461.54-613.36
		Cervical-axial	668.74 (55.39)^B^	587.72-747.95
		Middle-axial	933.65 (74.73)^C^	795.15-1041.24
		Occlusal	1618.56 (239.58)^D^	1358.85-2131.47

Different superscript uppercase letters (A, B, C, D) in the same column indicate statistically significant difference (*p* < 0.05). *μ*m: micrometer.

## Data Availability

All data of the present article are available on request by contacting the corresponding author.
